# Returnformer: A Graph Transformer-Based Model for Predicting Product Returns in E-Commerce

**DOI:** 10.3390/e28010072

**Published:** 2026-01-08

**Authors:** Qian Cao, Ning Zhang, Huiyong Li

**Affiliations:** 1 School of Computer and Artificial Intelligence, Beijing Technology and Business University, Beijing 100048, China; 2331102067@st.btbu.edu.cn; 2 School of Artificial Intelligence (Institute of Artificial Intelligence), Beihang University, Beijing 100191, China; lihuiyong@buaa.edu.cn

**Keywords:** return prediction, e-commerce, graph algorithm, topological structure

## Abstract

E-commerce retailers bear substantial additional costs arising from high product return rates due to lenient return policies and consumers’ impulsive purchasing. This study aims to accurately predict product return behavior before payment, supporting proactive return management and reducing potential losses. Based on the Graph Transformer, we proposed a novel return prediction model, Returnformer, which focuses on capturing user–product connections represented in topological structures of bipartite graphs. The Returnformer first integrates global topological embeddings into original node features to alleviate structural information loss caused by graph partitioning. It then employs a Graph Transformer to capture long-range user–item dependencies within local subgraphs. In addition, a graph-level attention mechanism is introduced to facilitate the propagation of global return patterns across different subgraphs. Experiments on a real-world e-commerce dataset show that the Returnformer outperforms four machine learning models in terms of prediction accuracy, demonstrating superior performance compared to the state-of-the-art models. The proposed model enables retailers to identify potential return risks prior to payment, thereby supporting timely and proactive preventive interventions.

## 1. Introduction

With the continuous updating of e-commerce platform technology, global online sales have been rising in recent years [1]. However, the product return rate has continued to rise due to a relaxed return policy [2], the discrepancy between advertising and the actual product [3], and delayed delivery [4]. This is especially obvious in the fashion industry, where the return rate ranges from 13% to 96%, with an average of 53% [5]. The high return rate has significantly eroded the profits of retailers. What’s more, product returns generate 2 to 16 times more greenhouse gases than the production and distribution of standard products [6]. Therefore, returns become a challenge to both the economy and ecology.

To effectively address the return problem, an in-depth understanding of customer behavior is essential, since return decisions are ultimately made by customers. Suppose retailers can predict the likelihood of product returns before customers pay, they may be able to take preventive measures to reduce this behavior, such as adjusting payment methods or offering reduced discounts. Therefore, a model that can estimate the probability of returns before payment can help retailers take timely measures to avoid the risk of returns.

Recent studies on forecasting return intention are mainly categorized into machine learning technology [7,8,9] and graph representation learning [10,11,12]. Compared with traditional machine learning, graph representation learning, which efficiently extracts complex potential patterns and relational features from data, performs satisfactorily in modeling users’ preferences [13]. However, due to the random division of the graph, the structural information of subgraphs becomes fragmented, which may hinder the transmission of information. Moreover, existing graph algorithms don’t make more use of the structural information of large-scale graphs. In addition, most present studies focus on the feature similarity between any customers and between any products, while ignoring the interrelationship between nodes in subgraphs, which represents the similarity of return patterns. To address these problems, we designed a novel return prediction model, called Returnformer, based on the Graph Transformer. The model works at the level of individual product variants. The main contributions of this study are summarized as follows:

1. We introduced Returnformer, a new return prediction model based on the Graph Transformer. By using the self-attention layer, it can effectively aggregate high-order information in the customer–product interaction graph. Furthermore, we have also introduced the Kolmogorov–Arnold Network (KAN) as a decoder to improve classification performance by constructing complex decision-making boundaries through nonlinear transformation.
2. The topological embeddings generated by Node2Vec are utilized to supplement the structural information. And then we adopt a dual-path feature fusion method, which combines the structural embedding with the original feature embedding, to obtain the final fusion embedding as the input feature.
3. We use the graph-level attention mechanism to capture the relations between customers and products in different subgraphs. This method enables similar nodes in different subgraphs to interact, thereby capturing the global characteristics and patterns of customer return behaviors.

The rest of this paper is as follows. Section 2 reviews the related works on the return of products in online shopping. Subsequently, the methodology of this paper is outlined in Section 3, which describes the dataset used and details the developed Returnformer. The validity of Returnformer is verified, and a discussion is provided in Section 4, followed by the conclusions and future works in Section 5.

## 2. Related Works

There has been extensive research on product returns. Some focus on exploring the factors affecting the return of products, such as consumer behavior [3,14,15], product characteristics [16,17,18,19], retailer reputation [20], logistics [21,22], and return policy [2]. Some even conduct pricing decision-making research on different return policies [23]. However, forecasting product returns is the key to this study. Therefore, this section reviews related studies based on their prediction targets and modeling approaches.

### 2.1. Prediction of Product Return Rate and Return Volume

Research on return rate and return volume prediction aims to forecast the overall return trend. This helps enterprises manage inventory and formulate production strategies. Dzyabura et al. [5] explored a predictive model that can forecast the product return rate based on the image before the product goes on the market. Rajasekaran and Priyadarshini [24] introduced product feedback scores and divided the return rate into five levels to help e-commerce platforms identify products with high return rates. However, for manufacturers, the return volume is more important because it has a significant impact on the production plan. Cui H et al. [25] used a LASSO model with main effects and interaction effects to forecast the future returns volume of automotive manufacturers.

Compared with traditional manufacturers, predicting the amount of returns in advance is more significant for remanufacturers since they need to make secondary use of returned products. Studies mentioned in [26,27] respectively adopted Grey-GERT and a dynamic prediction model selection algorithm to forecast the return of recyclable products, so as to improve the profitability of the remanufacturing industry. Chou et al. [28] further incorporated historical demand and sales data into their return forecasting model, supporting more adaptive remanufacturing strategies.

### 2.2. Prediction of Product Return Propensity

The forecast of the return volume and return rate usually focuses on the overall product return trend, rather than the forecast of a single product, and also, these predictions are made after purchase. Whereas consumers are often uncertain about whether to keep or return products. Previous studies tried to predict this uncertainty by predicting customers’ tendency to return. These studies can be roughly divided into two categories according to different strategies. The former adopts traditional machine learning to predict returns, while the latter focuses on using graph algorithms to predict the probability of returns.

#### 2.2.1. Traditional Machine Learning Models for Return Prediction

In terms of feature construction, Fu Y et al. [29] developed a general return tendency model by using the potential vector obtained from the decomposition of product returns to forecast the return tendency of consumers. Urbanke P [30] designed a feature extraction method for high-dimensional sparse data to achieve feature dimension reduction and predict product returns. In addition, Duong Q H et al. [31] mainly extracts the intrinsic and external attributes of the product from customer reviews to forecast the probability of returns. Notably, Hofmann A [32] proved that return behaviors can be effectively predicted based only on basic order characteristics such as product name, price, and quantity.

From a modeling perspective, some studies regard the problem of consumer returns as a second classification problem. By setting a threshold, they convert the predicted return probability into binary prediction results to forecast whether consumers will return the goods. These studies systematically compare various traditional machine learning models [7,8], and even evaluated the model performance under different optimizations [9] to balance predictive accuracy with interpretability.

#### 2.2.2. Leveraging Graph Representation for Return Prediction

The inherent structure of consumer-product interactions naturally exhibits graph-like characteristics. Therefore, there is a growing trend of research that explores the potential of graph representation learning on product returns. Different studies have modeled consumer purchase and return records using various graph algorithms, including hypergraphs [10], weighted graphs [11], heterogeneous graphs [12,33], and bipartite graphs [13,34,35].

Li et al. [10] and Zhu et al. [11] employed hypergraph and weighted hybrid graph modeling, respectively, to compute feature similarities and identify similar shopping baskets, customers, or products. They then utilized transition probabilities from random walk and its variant algorithms to forecast return likelihood, and further return behaviors. Similarly, Li et al. [12] incorporated order-user similarity into a trust network, then applied random walk algorithms for prediction.

In contrast, Joshi et al. [34] divided consumer-product interaction networks into various behavior communities via community detection, employing Support Vector Machines (SVM) for prediction. Kedia et al. [35] integrated industry-specific characteristics and users’ body shape by combining MF-BPR with Skip-gram models, and then deep neural networks were adopted to forecast returns. The similarity of these approaches is that they extract features before final classifying through predefined algorithmic rules, such as walking, partitioning, and similarity calculation.

The emergence of graph neural networks (GNNs) [36,37,38,39] has provided a new way to learn node representation, which can aggregate neighbor information through a message-passing mechanism to complete the update of node representation. McGowan et al. [13] demonstrated the superior performance of GNN for returns. On this basis, Ma and Wang [33] proposed an inductive HGNN that integrates user, product, and order features to construct a return prediction framework.

Although GNNs offer greater advantages over traditional graph algorithms, these approaches still have a deficiency of local information aggregation [40]. Furthermore, e-commerce interaction graphs are typically large-scale. To improve computing efficiency, this graph is usually randomly divided into countless subgraphs, which will lead to the destruction of global structural information. Additionally, these studies primarily focus on the features of individual nodes and neglect the correlation of different nodes between subgraphs. Table 1 compares the return prediction research using different graph representation methods.

Inspired by the performance of Graph Transformer [41] on large-scale graphs, we develop a new return forecasting model, Returnformer, based on the Graph Transformer to address these constraints. Our method explicitly integrates the embedding of topological structure. What’s more, it employs the graph-level attention mechanism [42] to capture the return pattern between different subgraphs. we ultimately introduce the Kolmogorov–Arnold Networks (KAN) [43] as a classifier to get the final return probability.

## 3. Methodology

We first introduce the dataset used in this study, along with the bipartite graph established herein. And subsequently, a detailed exposition of prediction model is provided.

**Figure 1:**
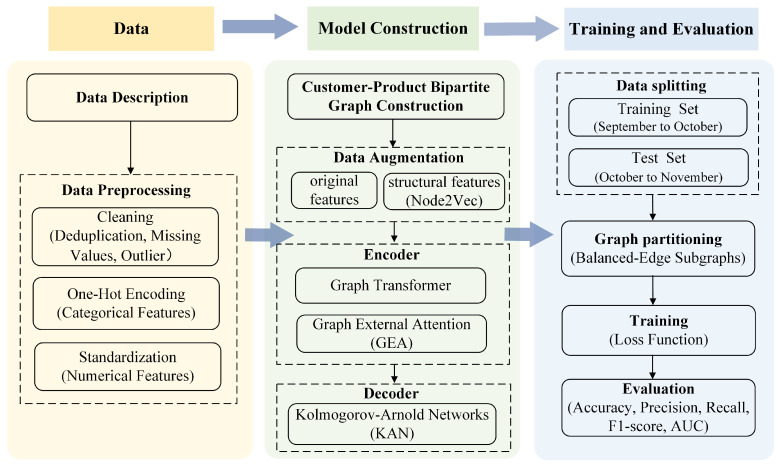
The research flowchart.

To better illustrate the research process, Figure 1 summarizes the main steps of this study. The process begins with data description and preprocessing, followed by the construction of a customer–product bipartite graph. Global structural features is extracted and fused with original features. And then the fused features are used as inputs to the proposed Returnformer for training. The model performance is finally evaluated on the test set using standard classification metrics.  

### 3.1. Data and Customer–Product Bipartite Graph Construction

#### 3.1.1. Data Description

Due to the high return rate of clothing products and the significant differences in their customer behaviors, this study utilizes the clothing dataset to construct the customer–product bipartite graph and verify our model. The dataset encompasses records of customer keep and return from September to November 2021, provided by the UK fast fashion e-commerce company ASOS. To prevent data leakage, these customers’ keep and return records are segmented by month. The training set encompasses historical data from September to October, while the test set covers records from October to November.

Both the training set and the test set contain three tables: an event table, a customer table, and a product table. These encompass not only approximately 1.8 million customer keep–return events, but also over 1.08 million unique users and more than 300,000 product variants. To protect privacy, all sensitive data is anonymized. We regard customer return prediction as a binary classification task. Returned events are labeled as 1, and kept events are labeled as 0. Each event in the dataset is marked accordingly.

#### 3.1.2. Data Preprocessing

We first checked and preprocessed the data, including identifying duplicates, missing values, and outliers. Then we deleted duplicates and recoded the customer ID and the product ID. Subsequently, the customer’s birth year is converted into age to enrich the user portrait. However, about 30,000 customers were over 90 years old, and these outliers were excluded to minimize errors. The final training set consisted of 939,537 events, while the test set contained 858,526 events. To match these events, there are 1,084,504 users and 338,076 product variants. We assessed the sample distribution of the final training and test sets. The ratio of positive to negative samples is approximately 1.2:1, indicating no significant class imbalance. Additionally, we performed one-hot encoding on categorical variables and standardized numerical variables to facilitate model training.

#### 3.1.3. Customer–Product Bipartite Graph Construction

Customer return behavior does not arise solely from isolated customer or product attributes, but from repeated interactions between customers and products over time. A customer might repeatedly purchase the same item or return multiple products. A product may also be purchased by multiple customers.

To preserve these interaction patterns, we construct a customer–product bipartite graph to represent the connection relationship between customers and items. Since customers and products are two types of entities with different attributes, we define them as two different types of nodes. Each type of node has its own attributes as shown in Table 2.

**Figure 2:**
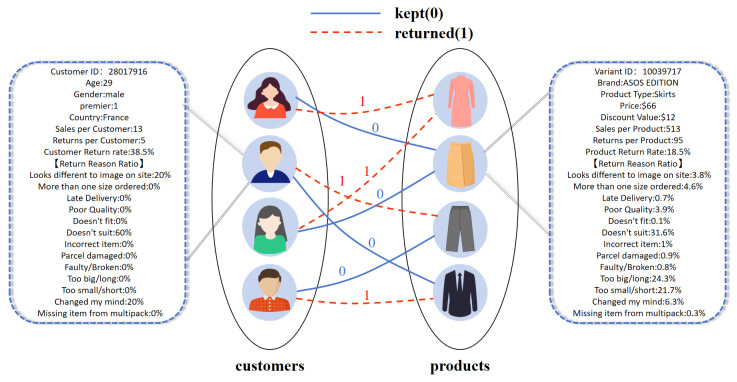
The customer–product bipartite graph.

The events occurring between customers and products are represented as edges connecting the corresponding nodes. In this study, there are two types of edges: returned edges and kept edges. They are marked as 1 or 0 respectively. This bipartite graph denoted as G=(U,I,E), where U,I, and *E* represent the sets of user nodes, product nodes, and edges, respectively. As a result, our return prediction task in the bipartite graph is an edge-level classification task. The customer–product bipartite graph is illustrated in Figure 2, which respectively display the specific values of a customer and a product.

### 3.2. Proposed Model

**Figure 3:**
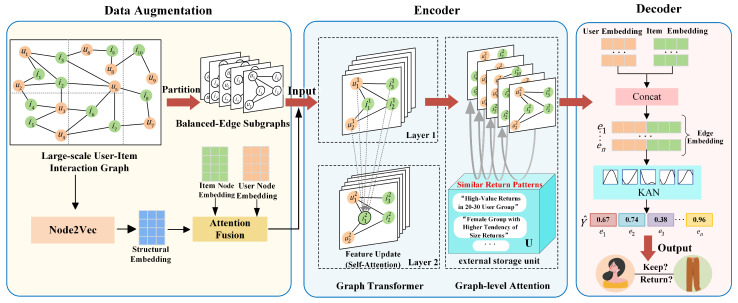
The Returnformer framework.

We propose the Returnformer, a return prediction model based on the Graph Transformer. The Returnformer framework is illustrated in Figure 3, which comprises three primary components: data augmentation, encoder, and decoder.

#### 3.2.1. Data Augmentation

The large-scale user–product interaction graph in e-commerce often comprises millions of nodes and edges. To make computation feasible, the graph is often partitioned into smaller subgraphs. However, this approach may disrupt the overall graph’s topological structure. For instance, when connections between high-return-rate products are fragmented across different subgraphs, this may potentially lead to the loss of critical relationships between users and products.

**Figure 4:**
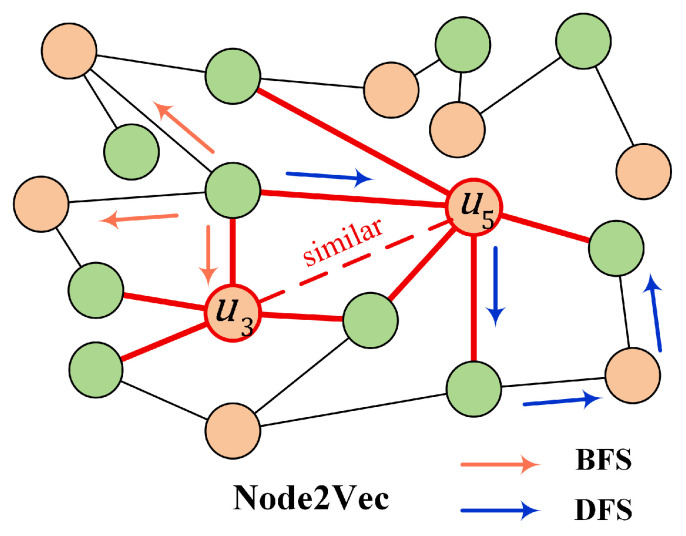
Node2Vec.

To address this problem, we leverage Node2Vec to obtain the global topological structure of the user–product interaction graph. Node2Vec controls breadth-first (BFS) and depth-first (DFS) search behaviors during random walks by adjusting hyperparameters *p* and *q*. It can capture the structural information and encode it into low-dimensional vectors. And the process is shown in Figure 4.

**Figure 5:**
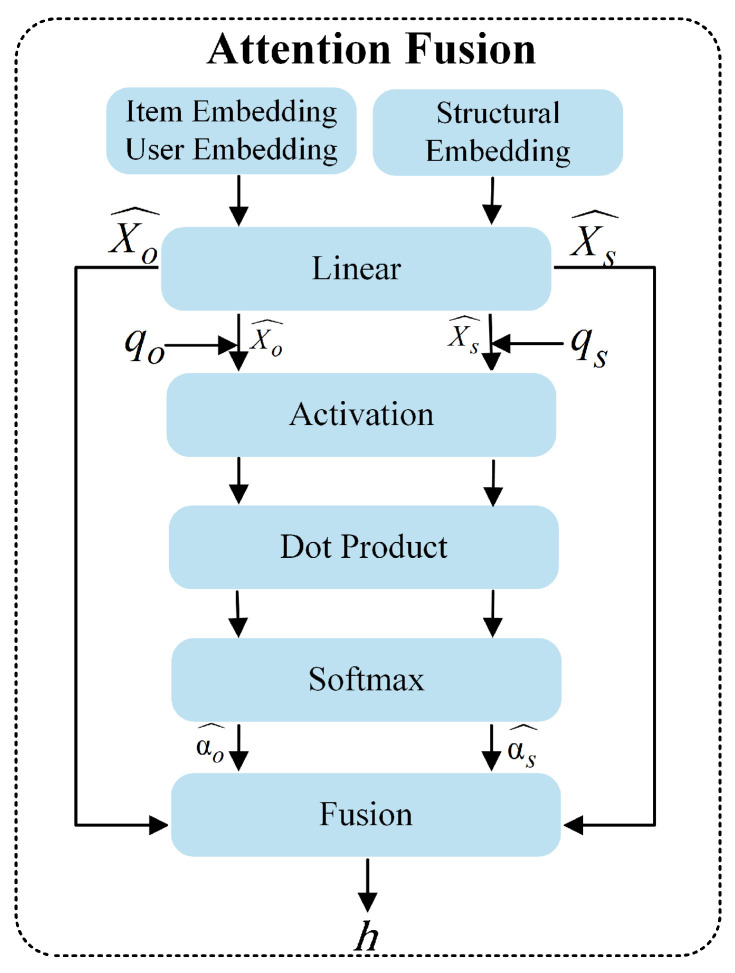
The attention feature fusion mechanism.

Meanwhile, we make use of an attention mechanism to fuse original and structural features dynamically. Unlike static fusion methods, which may obscure important features, our approach adaptively combines features based on their importance. The detailed fusion process is shown in Figure 5.

Formally, let $X_{o},X_{s}\in R^{N\times d}$ be the input features, *N* be the number of nodes, and *d* be the feature dimension. We first apply linear transformations:

(1) $$\left\{\begin{array}{c} \begin{array}{c} \hat{X_{o}} \end{array}=X_{o}W_{o}\\ \begin{array}{c} \hat{X_{s}} \end{array}=X_{s}W_{s} \end{array}\right.$$

where $W_{o},W_{s}\in R^{d\times d}$ are the learnable parameter. $X_{o}$ represents the original features, and $X_{s}$ denotes the structural features generated by Node2Vec.

Next, we compute attention weights for the original and structural features,

(2) $$\left\{\begin{array}{c} \alpha_{o}=\tanh\left(\begin{array}{c} \hat{X_{o}} \end{array}q_{o}\right)\\ \alpha_{s}=\tanh\left(\begin{array}{c} \hat{X_{s}} \end{array}q_{s}\right) \end{array}\right.$$

where $q_{o},q_{s}\in R^{d}$ are the attention vectors.

Furthermore, following softmax normalization, the attention weights are expressed as

(3) $$\left\{\begin{array}{c} \begin{array}{c} \hat{\alpha_{o}} \end{array}=\frac{\exp\left(\alpha_{o}\right)}{\exp\left(\alpha_{o}\right)+\exp\left(\alpha_{s}\right)}\\ \begin{array}{c} \hat{\alpha_{s}} \end{array}=\frac{\exp\left(\alpha_{s}\right)}{\exp\left(\alpha_{o}\right)+\exp\left(\alpha_{s}\right)} \end{array}\right.$$

Ultimately, the fused embedding *h* can be represented as

(4) $$\begin{array}{c} h=\hat{\alpha_{s}}\hat{X_{s}}+\hat{\alpha_{o}}\hat{X_{o}} \end{array}$$

#### 3.2.2. Encoder

The encoder learns representations from graph data by mapping nodes, edges, or the whole graph into a low-dimensional vector space. The encoder of this model primarily adopts a Graph Transformer and incorporates a graph-level attention mechanism to enhance the model’s expressive capacity.

(1) Graph Transformer

**Figure 6:**
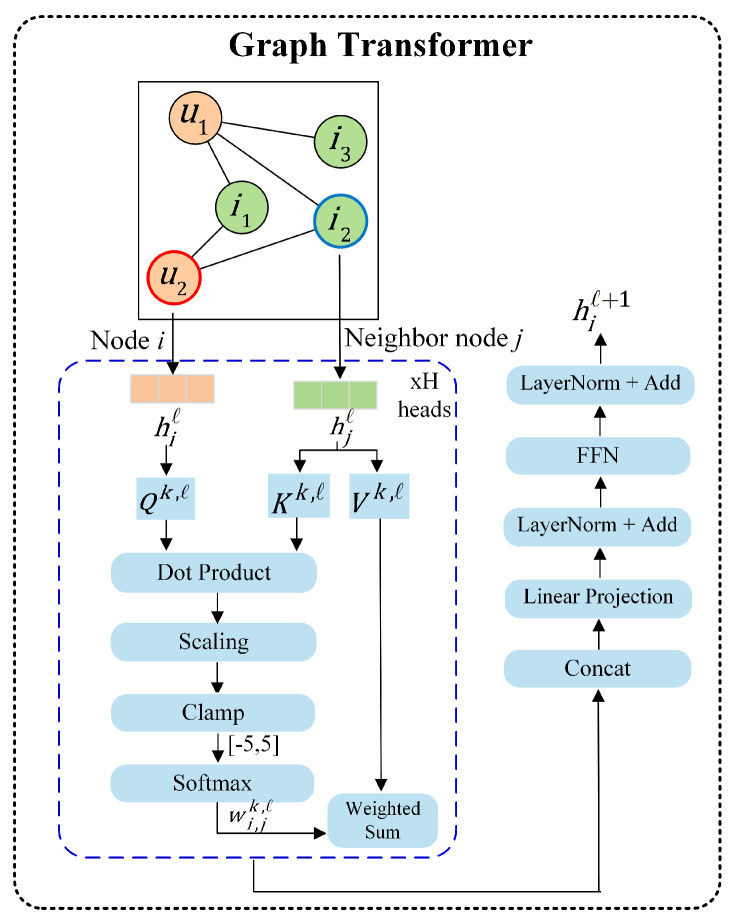
Graph Transformer.

The Graph Transformer uses self-attention to let each node interact with all other nodes in its subgraph. This captures long-range dependencies and allows user and product information to propagate globally within the current subgraph. The proposed model simplifies the Graph Transformer framework by removing the edge feature processing. Meantime, the above graph structure is integrated, which makes it more suitable for modeling sparse user–item interactions. Figure 6 illustrates the detailed process of Graph Transformer.

To obtain the node features of the updated (*ℓ* + 1)-th layer, we need to use Equation (5) to calculate Q,K, and *V* of each attention head in the *ℓ*-th layer,

(5) $$\left\{\begin{array}{c} Q_{i}^{k,\ell }=Q^{k,\ell }h_{i}^{\ell }\\ K_{j}^{k,\ell }=K^{k,\ell }h_{j}^{\ell }\\ V_{j}^{k,\ell }=V^{k,\ell }h_{j}^{\ell } \end{array}\right.$$

where $Q^{k,\ell },K^{k,\ell },V^{k,\ell }\in R^{d_{k}\times d}$ are the learnable weight matrices, and $h_{i}^{\ell },h_{j}^{\ell }$ are the input features of the computing node *i* and *j* its neighbor nodes at the *ℓ*-th layer, respectively. The fusion features *h* from Equation (4) serves as the initial input of the 0-th layer.

Subsequently, the attention weight of a node *i* to its neighbor nodes $j\in N_{i}$ can be expressed as

(6) $$w_{ij}^{k,\ell }=\mathrm{softmax}{\left(\frac{Q_{i}^{k,\ell }\cdot K_{j}^{k,\ell }}{\sqrt{d_{k}}}\right)}^{T}$$

where $d_{k}$ represents the dimension of each head. To ensure numerical stability, the input of Softmax is clamped to the range of −5 to +5.

After obtaining the attention weights for each head, all the matrices are concatenated and processed through the output projection matrix $O_{h}^{\ell }$ to obtain the multi-head attention output ${\hat{h}}_{i}^{\ell +1}$. This process is implemented by Equation (7),

(7) $${\hat{h}}_{i}^{\ell +1}=O_{h}^{\ell }{∥}_{k=1}^{H}\left(\sum_{j\in N_{i}}w_{ij}^{k,\ell }V^{k,\ell }h_{j}^{\ell }\right)$$

where the range of *k* is from 1 to *H*, and *k* represents the number of attention heads, $O_{h}^{\ell }\in R^{d\times d}$.

Finally, through two normalizations and residual connections, as well as a two-layer feedforward network, the updated node features $h_{i}^{\ell +1}$ are obtained as follows,

(8) $${\tilde{h}}_{i}^{\ell }=\mathrm{LayerNorm}\left(h_{i}^{\ell }\right)+{\hat{h}}_{i}^{\ell +1}$$

(9) $${\bar{h}}_{i}^{\ell }=W_{2}^{\ell }\mathrm{GELU}\left(W_{1}^{\ell }{\tilde{h}}_{i}^{\ell }\right)$$

(10) $$h_{i}^{\ell +1}=\mathrm{LayerNorm}\left({\tilde{h}}_{i}^{\ell }\right)+{\bar{h}}_{i}^{\ell }$$

where $W_{1}\in R^{d_{\mathrm{ff}}\times d},W_{2}\in R^{d\times d_{\mathrm{ff}}}$ are the weight matrices, and $d_{\mathrm{ff}}$ is the hidden dimension of the feed-forward network.

(2) Graph-level Attention Mechanism

We partition the large interaction graph into several subgraphs with the same number of edges. The Graph Transformer encode within a single subgraph, ignoring the potential correlations between subgraphs. For instance, users A and B who belong to different subgraphs may exhibit similar behaviors, such as frequently returning the same type of goods.

To address this, we introduce a graph-level attention mechanism to enhance the Graph Transformer, which called Graph External Attention (GEA). This mechanism integrates information from external graphs into the current graph’s representation learning. It enhances the model’s ability to capture both local embeddings and global inter-graph correlations. The GEA computes attention weights from node features in the current graph to external key-value units by Equations (11) and (12). Algorithm 1 demonstrates the pseudo-training algorithm of GEA.
**Algorithm 1** Framework of the GEA**Input:** Graph with node set *V*, node embeddings *X***Output:** Updated node representations $X_{\mathrm{out}}$  1: Initialize model parameters: external key-value units $U_{k}$, $U_{v}$, and number of heads $H_{ext}$  2: **for** each node ∈X **do**  3:    $X_{\mathrm{reshaped}}=\mathrm{Reshape}\left(X,\left(n,\begin{array}{c} H_{ext} \end{array},d/\begin{array}{c} H_{ext} \end{array}\right)\right)$  4:    $Q=X_{\mathrm{reshaped}}U_{k}^{T}$  5:    A=DNorm(Q)  6:    $X_{\mathrm{attn}}=AU_{v}$  7:    $X_{\mathrm{out}}=\mathrm{Reshape}\left(X_{\mathrm{attn}},\left(n,d\right)\right)$  8: **end for**  9: **return** $X_{\mathrm{out}}$

The attention matrix $A_{ext}$ is obtained by two steps. First, matrix multiplication on the node embedding matrix $X\in R^{n\times d}$ and a learnable parameter matrix $U_{k}\in R^{m\times d}$ is performed. Then, dual normalization technique DNorm(·) is applied:

(11) $$A_{ext}=DNorm\left(XU_{k}^{T}\right)$$

where $U_{k}$ can be regarded as an external key storage unit containing *m* virtual nodes. The similarity between the current node and the *m* virtual nodes in the external unit is calculated. When computing the attention matrix $A_{ext}$ for the first time, we use the node features $h_{i}^{\ell +1}$ updated by Equation (10) as the node feature matrix *X*.

The dual normalization first computes the similarity ${\tilde{S}}_{i,j}$ between nodes *i* and neighbor nodes *j* through Equation (12).

(12) $${\tilde{S}}_{i,j}={\left(XU^{T}\right)}_{i,j}$$

Then Equations (13) and (14) are used to normalize each column and each row, respectively, to improve the stability of the values.

(13) $${\hat{S}}_{i,j}=\frac{\exp\left({\tilde{S}}_{i,j}\right)}{\sum_{k=0}^{n}\exp\left({\tilde{S}}_{k,j}\right)}$$

(14) $$\alpha_{i,j}=\frac{{\hat{S}}_{i,j}}{\sum_{k=0}^{S}{\hat{S}}_{i,k}}$$

The final node features are obtained by multiplying the normalization attention matrix and the external value unit $U_{v}$, as demonstrated in Equation (15).

(15) $$X_{out}=A_{ext}U_{v}$$

#### 3.2.3. Decoder

The decoder uses the encoded embeddings for link prediction. Specifically, the Kolmogorov–Arnold Networks (KAN) is introduced as the decoder. Traditional GNNs typically employ inner products or shallow multilayer perceptrons (MLPs) for decoding. Whereas, these methods not only make it difficult to model complex decision boundaries in user–product interactions, but also result in a substantial number of parameters.

**Figure 7:**
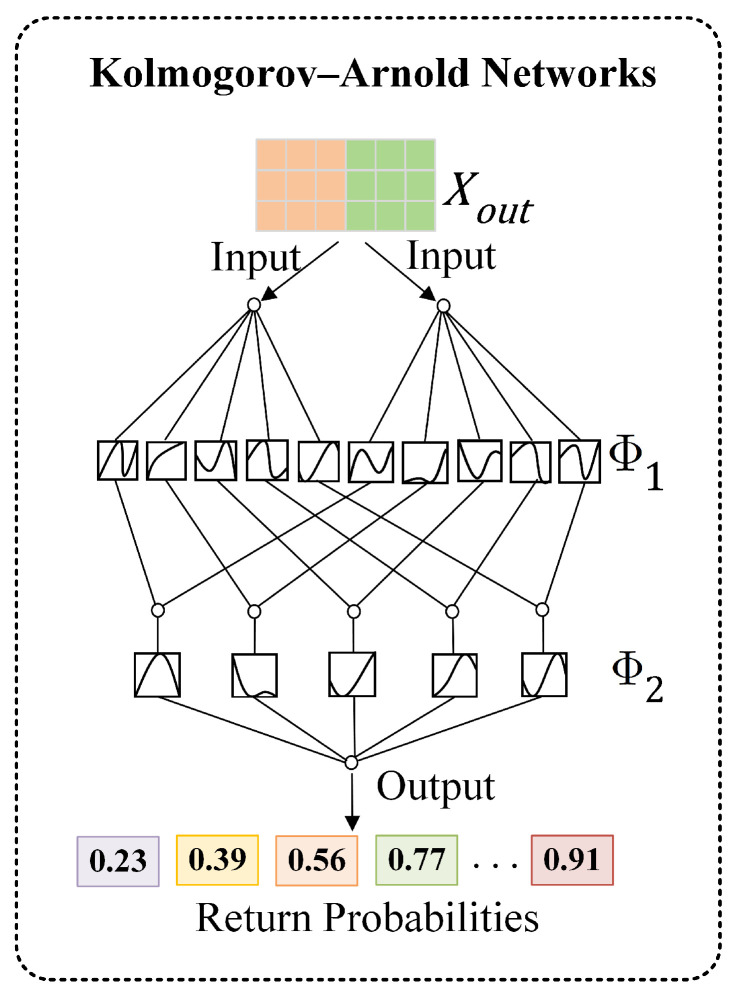
Kolmogorov–Arnold Networks.

In contrast, the KAN adaptively learns nonlinear feature relationships via B-spline functions. Specifically, each learnable function is obtained by combining different parameters of B-splines. By placing these functions on edges rather than nodes, the KAN captures complex interaction patterns with fewer parameters. Figure 7 illustrates its application in our model.

Equations (16) and (17) detail MLP, while Equations (18) and (19) describe the KAN. MLP achieves nonlinear mapping by repeatedly applying linear transformations using fixed activation functions, described as follows:

(16) $$\mathrm{MLP}\left(X_{out}\right)=\left(F_{L}\circ F_{L-1}\circ \cdots \circ F_{1}\right)\left(X_{out}\right)$$

The transformation of its *ℓ*-th layer is shown in Equation (17), which consists of the weight matrix $W_{\ell }$, the bias $b_{\ell }$, and the activation function σ.

(17) $$F_{\ell }\left(z\right)=\sigma \left(W_{\ell }z+b_{\ell }\right)$$

The KAN defines each layer as a trainable univariate function matrix, which can be expressed as

(18) $$\mathrm{KAN}\left(X_{out}\right)=\left(\Phi_{L}\circ \Phi_{L-1}\circ \cdots \circ \Phi_{1}\right)\left(X_{out}\right)$$

$\Phi_{\ell }$ is the function matrix of the *ℓ*-th layer, as shown in Equation (19). $\phi_{\ell ,p,q}$ is the trainable univariate function from the *p*-th input to the *q*-th output in the *ℓ*-th layer,

(19) $$\Phi_{\ell }=\left\{\phi_{\ell ,p,q}\right\},p=1,\cdots ,M_{in}^{\left(\ell \right)},q=1,\cdots ,M_{out}^{\left(\ell \right)}$$

where, $M_{in}^{\left(\ell \right)}$ and $M_{out}^{\left(\ell \right)}$ are the input dimension and the output dimension in the *ℓ*-th layer, respectively.

The above demonstrates the specific processes of each part of the Returnformer. We outline the pseudo-training algorithm of Returnformer using Algorithm 2.
**Algorithm 2** Framework of Returnformer**Input:** User–item bipartite graph G=(U,I,E); user features $X_{u}$; item features $X_{i}$; Node2Vec embeddings $X_{n2v}$**Output:** Edge-level prediction return probabilities $\hat{Y}$  1:  Initialize model parameters θ randomly  2:  Feature fusion: $X_{f}=\mathrm{Attention}\mathrm{Fusion}\left(X_{u},X_{i},X_{n2v}\right)$  3:  Initialize node embeddings: $H^{\left(0\right)}=\left[X_{fu}∥X_{fi}\right]$  4:  **for** each edge $\in H^{\left(0\right)}$ **do**  5:     $V_{u}=X_{fu}\left[u_{\mathrm{idx}}\right],V_{i}=X_{fi}\left[i_{\mathrm{idx}}\right]$  6:     $H_{u}^{l+1},H_{i}^{l+1}=\mathrm{Graph}\mathrm{Transformer}\left(V_{u},V_{i}\right)$  7:     $H_{u}^{\mathrm{out}},H_{i}^{\mathrm{out}}=\mathrm{GEA}\left(H_{u}^{l+1},H_{i}^{l+1}\right)$  8:     $X_{\mathrm{out}}=\left[H_{u}^{\mathrm{out}}∥H_{i}^{\mathrm{out}}\right]$  9:     $\hat{Y}=\mathrm{KAN}\left(X_{\mathrm{out}}\right)$10:  **end for**11:  **return** $\hat{Y}$

## 4. Experiments

In this section, we evaluate our proposed model using the processed return data. The experiments are implemented in Python 3.9, utilizing open-source libraries such as sklearn, pandas, and numpy.

### 4.1. Experimental Environment Setup

We employed the original distribution of the training set and the test set to develop and evaluate our Returnformer. Due to the limitations of the GPU, nearly one million events were divided into multiple batches for training. We set the return probability threshold to 0.5 and use the cross-entropy loss function to quantify the difference between the predicted value and the actual value. The model is optimized utilizing the Adam optimizer and the automation method Optuna, which helps us automatically search for the best hyperparameters and quickly converge to the minimum value of the loss function. Specifically, the search ranges for batch size and embedding dimension are set to [64, 128, 256] and [16, 32, 64, 128, 256], respectively. The learning rate and dropout rate are searched within the ranges of [1 × 10^−5^, 1× 10^−3^] and [0.2, 0.5]. The validation set is obtained by randomly sampling 10% from the training set to evaluate different hyperparameters. Finally, the parameter configuration used in this study is presented in Table 3.

### 4.2. Comparison Results

Four machine learning models and three graph neural network models were used as comparison models to assess the performance of Returnformer. These models include Multi-Layer Perceptron (MLP), Extreme Gradient Boosting (XGBoost), Light Gradient Boosting Machine (LightGBM), Categorical Boosting (CatBoost), Graph Convolutional Network (GCN), Graph Attention Network (GAT), and Graph Sample and Aggregate (GraphSAGE). Besides, we employed multiple metrics, including accuracy, precision, recall, F1-score, and area under the Receiver Operating Characteristic curve (AUC), to evaluate the model’s performance.

**Figure 8:**
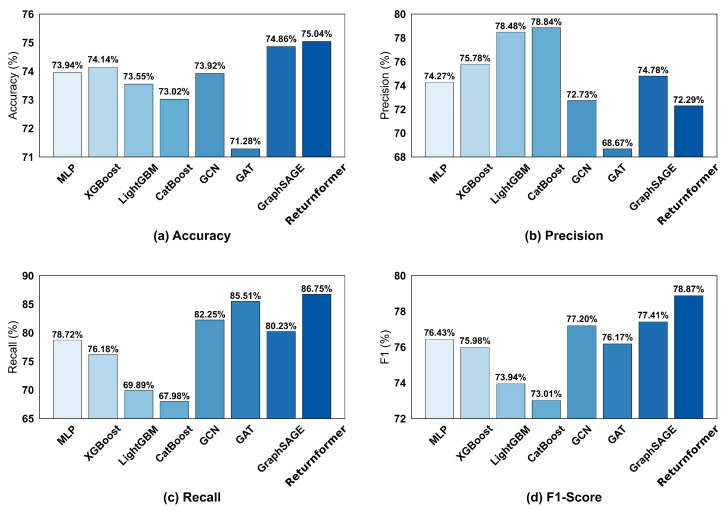
All comparison results on the test set. (**a**) Accuracy. (**b**) Precision. (**c**) Recall. (**d**) F1-score.

Figure 8 presents the performance metrics for all models on the dataset. The Returnformer surpassed all baselines in all metrics except precision. It achieved a recall of 86.75% and an F1-score of 78.87%, indicating that it has pleasant performance.

**Figure 9:**
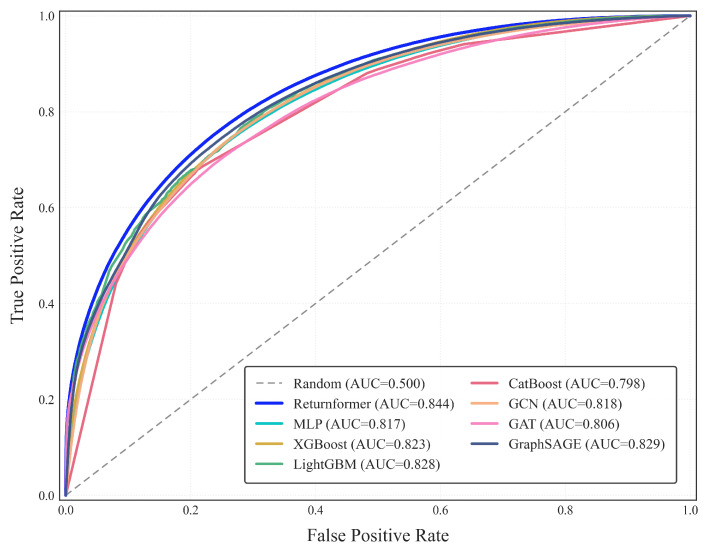
Receiver Operating Characteristic (ROC) curves.

The Receiver Operating Characteristic (ROC) curves of all models are displayed in Figure 9. The closer AUC is getting to 1, the stronger the model’s classification capability will be. The ROC curve of the Returnformer is closest to the upper left corner, with an AUC of 0.844, indicating that its classification ability surpass that of other models. Moreover, GNNs significantly outperformed machine learning models. Notably, the F1-score for GraphSAGE is 77.41%, which is the highest among all comparison models. This highlights the advantage of the graph representation learning method in forecasting user–item interaction behaviors.

Generally, customers with higher return rates tend to have higher expectations for products and are more likely to abuse the return policy [12]. To further validate the Returnformer’s capability in modeling users with high return rates, we conducted an additional experiment on the same training set. However, the scope of the test set is different. It includes only events from users with a return rate of 50% or higher. This specific test set can make a more rigorous assessment of the Returnformer.

As shown in Table 4, the Returnformer achieved an F1-score of 87.63% on this specialized test set, which is 8.76% higher than its F1-score on the entire test set. On the one hand, this displays that the Returnformer can make more accurate predictions about the return behavior of users with high return rates. On the other hand, it demonstrates the ability of the model to predict the return behavior of special users.

### 4.3. Ablation Analysis

We conducted ablation experiments by systematically removing each component to assess its contribution in the Returnformer after comparative experiments.

(1) *w*/*o* data augmentation: We eliminated the data augmentation component, meaning that the model only relies on the original feature of nodes without integrating the global structural information. (2) *w*/*o* graph-level attention: We removed the graph-level attention mechanism that captures the inter-graph correlations. Consequently, after two layers of Graph Transformer encoding, the feature is directly partitioned and concatenated. (3) *w*/*o* KAN: We omitted the KAN decoder from the model, replacing it with a single-layer linear classifier for the features after splicing.

**Figure 10:**
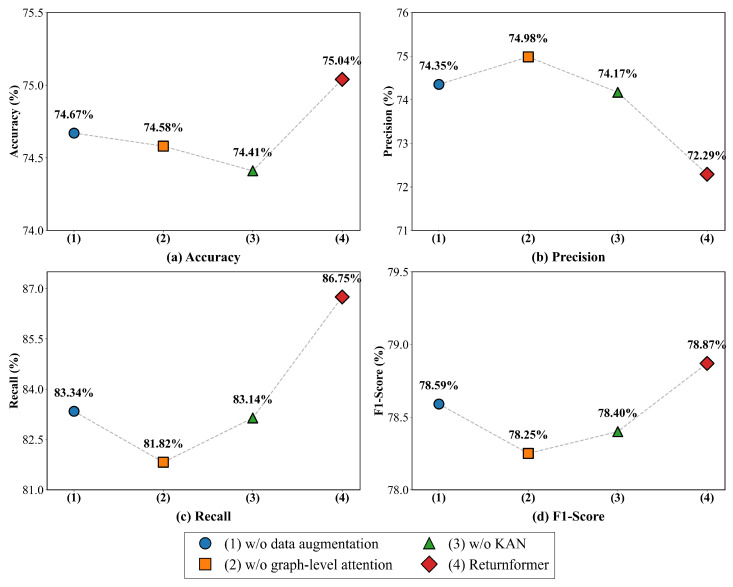
Results of Ablation Experiments.(**a**) Accuracy. (**b**) Precision. (**c**) Recall. (**d**) F1-score.

The results of ablation experiments are presented in Figure 10. Except for precision, other metrics of Returnformer perform better than those of all simplified versions. Each component plays the essential role in improving the ability to return predictions.

The data augmentation integrates the global topological structure into the original features, effectively compensating for the structural information lost during graph partition. This enhances the model’s representation ability. Additionally, the graph-level attention mechanism automatically learns the potential connections between subgraphs. This mechanism allows the model to learn the return patterns in subgraphs and apply them to the node representation in the new subgraph. Moreover, with its strong nonlinear fitting ability, the KAN accurately models complex decision-making boundaries in user–product interactions, which contributes to the final classification.

### 4.4. Sensitivity Analysis

To further verify the robustness of the proposed model, we conducted a sensitivity analysis on several key hyperparameters, mainly including the number of layers and the number of attention heads in the Graph Transformer, as well as the number of attention heads in the GEA. These parameters are critical since they are located in the encoding layers and directly control the model’s ability to capture high-order information and propagate information across subgraphs.

**Figure 11:**
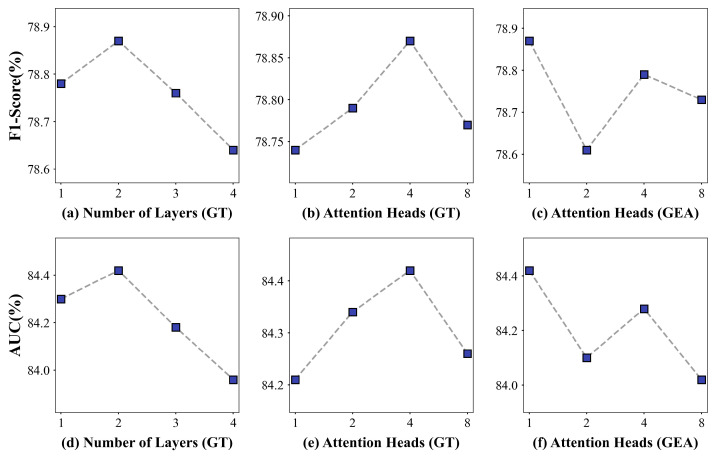
The comparison of F1-score and AUC under Different Configurations. (**a**) F1-Score of Graph Transformer(GT) with different numbers of layers; (**b**) F1-Score of Graph Transformer with different numbers of attention heads; (**c**) AUC of GEA with different numbers of attention heads; (**d**) AUC of Graph Transformer with different numbers of layers; (**e**) AUC of Graph Transformer with different numbers of attention heads; (**f**) F1-Score of GEA with different numbers of attention heads.

Specifically, the number of Graph Transformer layers varied from 1 to 4, while the number of attention heads in the Graph Transformer was set to 1, 2, 4, 8. In addition, we evaluated the GEA with 1, 2, 4, and 8 attention heads. For each configuration, the model was trained under the same experimental settings described in Section 4.1, and all configurations were evaluated using the same test set. The corresponding F1-score and AUC values under different configurations are illustrated in Figure 11.

It can be observed that the model performs stably across different hyperparameter settings, demonstrating the effectiveness of the proposed model. When the number of Graph Transformer layers exceeds two, the performance gradually degrades, which may be attributed to overfitting. Moreover, when the number of attention heads increases beyond four, redundancy in attention computation may occur. As the number of attention heads in the GEA varies, both the F1-score and AUC first decrease, then increase, and finally decrease again. Overall, this sensitivity analysis confirms that the proposed model is robust to appropriate variations in key hyperparameters. It also suggests that the predictive performance is mainly determined by the model architecture and input features, rather than strongly relying on specific hyperparameter settings.

### 4.5. Discussion

This study aims to forecast customers’ return risk in advance by learning from their historical transaction data. The comparison results in Figure 8 demonstrate that the Returnformer outperforms seven baseline models. Moreover, the experimental results also indicate that, compared to traditional machine learning methods, graph representation learning has stronger learning and prediction capabilities for data with natural graph structures. This is consistent with previous research [13], as graph representation learning is easier to learn complex patterns in the data, especially in this type of user–item interaction graph. The predictive results for users with a high return rate, presented in Table 4, demonstrate that the model we proposed is better suited for such users.

The results of the ablation experiments in Figure 10 also further confirm the validity of each component of the model. The topology structure in the user–item interaction graph may contain predictive information beyond the original node features. Additionally, the graph-level attention mechanism can adequately catch the commonality of users’ return patterns across different subgraphs, making an apparent contribution to return prediction. Figure 11 illustrates that the proposed model maintains stable performance under different hyperparameter settings, indicating good robustness.

**Figure 12:**
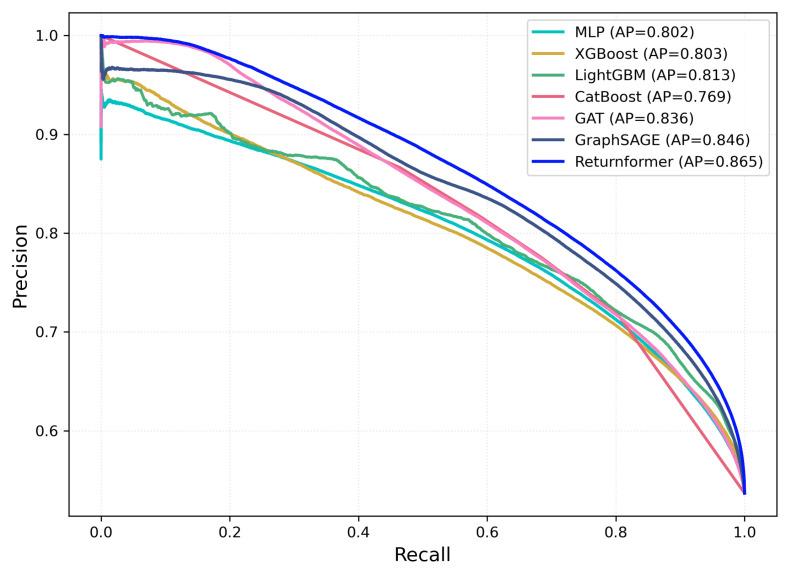
Precision–Recall(PR) Curves on the Test Set.

Through the analysis of various experiments, we found that the recall of our model is obviously higher than its precision. The Returnformer can perceive consumers’ return tendency more comprehensively. Although its precision is not ideal, this may lead the model to misclassify some low-risk customers as high-risk. However, return prediction is a risk-sensitive task, where failing to identify a true return customer generally incurs a higher operational cost than incorrectly labeling a low-risk customer. Moreover, the model outputs a continuous return probability, while the precision and recall reported in our experiments are calculated using a fixed decision threshold of 0.5. To further examine the trade-off between precision and recall of the proposed model, we plot the precision-recall(PR) curves of all models in Figure 12. The PR curves illustrate the performance of different models in terms of precision and recall across varying decision thresholds.

As shown in Figure 12, compared with other baseline models, the proposed Returnformer achieved higher precision at the same recall level. The Average Precision (AP) of our model reach 0.865, indicating that the model prioritizes recall under the default threshold and aims to capture users with potential return risk as comprehensively as possible. In practical e-commerce scenarios, return prediction models are typically used as decision-support tools rather than directly triggering punitive actions for all high-risk users. Therefore, retailers can flexibly adjust the decision threshold according to their tolerance for misclassification. By selecting an appropriate decision threshold, retailers can introduce preventive measures for users with a high predicted return risk, such as displaying reminder pop-ups or providing enhanced size recommendation services. At the same time, the implementation of these preventive strategies can also help retailers identify and filter users who tend to abuse return policies.

## 5. Conclusions and Future Works

This paper proposed a Graph Transformer-based return prediction model, Returnformer, which forecasts customers’ return tendencies before their payment. Based on the ASOS clothing dataset, our model achieved a recall of 86.75% and an AUC of 84.42%. In comparison with previous research methods, the Returnformer leverages the structural information of the user–item interaction graph and utilizes a graph-level attention mechanism to capture and disseminate global return patterns. As a result, the model exhibits robust performance in predicting return risk at the level of individual product variants, providing retailers with a tool for early warning of return risks.

Retailers can adopt proactive strategies based on forecasting models, such as personalized information reminders and adjusting freight. Retailers can also provide personalized informational prompts to users with a high predicted return risk, such as size recommendations and more detailed product information. Adjusting the freight will also prompt customers with a high return rate to evaluate the necessity of their purchase behavior more carefully. Meanwhile, the model also helps optimize the supply chain and improve profitability.

Although this study offers the advantages mentioned above, it still presents limitations. The model primarily relies on the keep–return events of users and products, as well as basic attributes of users and products. However, other factors may also influence return behaviors, such as product reviews, seasonal factors, and promotions. In addition, model predictions are usually based on learning from static historical data, yet user preferences and product popularity are dynamic. Applying real-time data to return prediction models remains a challenge. Future research will focus on developing a return prediction model that incorporates dynamic data learning and enhances the model’s interpretability.

## Figures and Tables

**Table 1:** The comparison of different studies on return prediction using graph representation methods.

Studies	Method	Comparison of Predictive Method Considerations			
		Similarity	Topological Structure	Graph Partitioning	Inter-Graph Correlation
Li et al. [10]	random walks	A truncated random walk	–	Local graph partitioning	–
Zhu et al. [11]	A random walk-based local algorithm	Hybrid similarity	–	LoGraph algorithm	–
Li et al. [12]	A trust-aware random walk model	Enhanced Pearson similarity	–	–	Trust-aware subgraph fusion
Ma and Wang [33]	Heterogeneous Graph Neural Network	–	Message passing	Heterogeneous graph sampling algorithm	–
Joshi et al. [34]	community detection +SVM	–	–	–	Multi-view subgraph fusion
Kedia et al. [35]	MF–BPR + skip-gram	–	–	–	–
McGowan et al. [13]	Graph Neural Network	–	Message passing	–	–
This study	Returnformer	Topological interaction similarity	Node2Vec to data Augmentation	Balanced-edge partitioning	Graph-level Attention Mechanism

**Table 2:** The node attributes of the bipartite graph.

Entity	Type	Attributes
customer	node	customer ID, age, gender, country, membership status, historical purchase volume, historical return volume, user return rate, the proportion of different return reasons
product	node	variant ID, brand, product type, average product price, average discounted product price, product sales volume, product return volume, product return rate, the proportion of different return reasons

**Table 3:** The parameter settings used in the proposed model.

Parameter	Definition	Setting
*p*	return parameter in Node2Vec	0.8
*q*	in-out parameter in Node2Vec	0.8
*ℓ*	number of layers in the Graph Transformer	2
*k*	number of attention heads in the Graph Transformer	4
*m*	number of virtual nodes in the memory unit of the GEA	20
$H_{\mathrm{ext}}$	number of attention heads in the GEA	1
*d*	embedding dimension	128
lr	learning rate	$2\times 10^{-5}$
dropout	dropout rate	0.45
batch_size	batch size	128

**Table 4:** The comparison results on the test set of users with high return rates (return rates ≥50%).

Model	Accuracy	Precision	Recall	F1-Score	AUC
Returnformer (all tests)	75.04%	72.29%	86.75%	78.87%	84.42%
Returnformer (high-return customers)	79.23% **(+4.19%)**	80.80% **(+8.51%)**	95.71% **(+8.96%)**	87.63% **(+8.76%)**	78.21%

The bold numbers indicate the improvement in Returnformer’s metrics on the high-return customers subset.

## Data Availability

The original data used in this study are openly available in the Open Science Framework (OSF) repository at https://osf.io/c793h/overview (accessed on 17 March 2025).

## Abbreviations

GNN	Graph Neural Network
GCN	Graph Convolutional Network
GAT	Graph Attention Network
GraphSAGE	Graph Sample and Aggregate
MLP	Multi-Layer Perceptron
XGBoost	Extreme Gradient Boosting
KAN	Kolmogorov–Arnold Networks
GEA	Graph External Attention
AUC	Area Under the Receiver Operating Characteristic Curve
ROC	Receiver Operating Characteristic
